# Sustaining allied health telehealth services beyond the rapid response to COVID-19: Learning from patient and staff experiences at a large quaternary hospital

**DOI:** 10.1177/1357633X211041517

**Published:** 2021-11-02

**Authors:** Michelle Cottrell, Clare L Burns, Amber Jones, Ann Rahmann, Adrienne Young, Sonia Sam, Mark Cruickshank, Kelsey Pateman

**Affiliations:** 1 3883 Royal Brisbane and Women's Hospital, Metro North Health, Australia; 2 School of Health and Rehabilitation Sciences, 1974 University of Queensland, Australia; 3 School of Allied Health, 95583 Australian Catholic University, Australia

**Keywords:** Allied health, telehealth, COVID-19, implementation, sustainability, quaternary care

## Abstract

The patient, clinician and administration staff perspectives of telehealth (specifically videoconferencing) services provided by Allied Health Professions (AHP) at a large quaternary hospital were explored. The purpose was to understand stakeholders’ perceptions of the service during initial COVID-19 restrictions and examine factors that influenced the implementation and sustained use of telehealth. A sequential mixed-methods approach was undertaken. Stage 1 involved surveys completed by patients (n = 109) and clinicians (n = 66) who received and provided care via telehealth, respectively, across six AHP departments. Stage 2 involved focus groups with clinicians (n = 24) and administrative staff (n = 13) to further examine implementation and sustainability factors.

All participant groups confirmed that telehealth was a valid service model and valued the benefits it afforded, particularly during COVID-19 restrictions. Both patients and clinicians reported that not all AHP services could be delivered via telehealth and preferred a blended model of telehealth and in-person care. Increased administrative staff assistance was needed to support growing telehealth demand. Main factors to address are the need to expand AHP telehealth models and workforce/patient training, improve workflow processes and enhance technical support.

Despite rapid implementation, telehealth experiences were overall positive. Study findings are being used to generate solutions to enhance and sustain AHP telehealth services.

## Introduction

On 23 March 2020, a series of measures, including significant physical distancing restrictions, were enacted by the Australian government to limit the spread of the SARS CoV-2 (COVID-19) virus. Almost overnight healthcare organisations had to alter the way in which individuals could access healthcare interventions, which led to the rapid and widespread implementation of telehealth. Despite the reported benefits of telehealth models, including high patient satisfaction, equivalent clinical outcomes and cost-effectiveness,^1–6^ its adoption into mainstream clinical practice had been slow prior to the COVID-19 pandemic.^7,8^ Much of the literature investigating this highlights that while adoption can be hindered by access to technological infrastructure,^7,9^ acceptance by front-line staff ultimately determines the success or failure of implementation efforts.^10,11^ As such, key practicalities need to be met when establishing a telehealth service, including telehealth platform selection, environmental set-up, patient selection and suitability, as well as ethical, operational and professional considerations.^12^ It is recommended that training and upskilling, irrespective of clinical experience, are undertaken to enable clinicians to provide safe and effective consultations via telehealth.^13,14^

As a metropolitan quaternary hospital, the Royal Brisbane and Women’s Hospital (RBWH) (Queensland, Australia) employs more than 400 allied health (excluding pharmacy) clinicians across nine professions, who offered more than 98,000 outpatient consults in 2019. While telehealth (specifically videoconferencing) had been successfully established within some clinical services, this activity only constituted 2.4% of all outpatient activity in 2019.^15^ The need to rapidly upscale telehealth across multiple clinical areas in response to COVID-19 meant that many of the recommended steps for implementing telehealth services could not be followed.^16,17^ This resulted in variable uptake and adoption of telehealth across Allied Health Professions (AHP). The control of COVID-19 within our jurisdiction enabled the easing of mandated restrictions to in-person consultations from July 2020. Despite this, community outbreaks continue, so there are organisational and societal incentives to embed telehealth as a part of standard clinical care delivery. With this in mind, the aim of this study was to (a) explore stakeholder perspectives towards the delivery of AHP healthcare via telehealth during COVID-19 and (b) examine the factors influencing implementation to inform the enhancement and sustainability of AHP telehealth services.

## Methods

### Study design and participants

A multi-stakeholder sequential explanatory mixed-methods approach was undertaken in two stages. Six AHP departments participated in this study, namely, Nutrition & Dietetics, Occupational Therapy, Physiotherapy, Psychology, Social Work and Speech Pathology. Stage 1 involved study-specific surveys completed by patients and AHP clinicians (here on referred to as ‘clinician’). Stage 2 incorporated interviews/focus groups with clinicians and administration officers (AO) employed in the AHP service. Eligible Stage 1 participants included any (a) patient (≥16 years) who had received, or (b) clinician who had provided, outpatient care via telehealth from the RBWH during the mandated COVID-19 restrictions (March–June 2020 inclusive). To avoid the potential for confounding responses, only those patients who received services from a single AHP department via telehealth (and who did not access any medical services via telehealth during this time) were considered for inclusion. Sample size calculations, using a 95% confidence interval and 5% margin of error, identified a minimum sample of 285 patient participants.^18^ However, to ensure adequate representation across all AHP departments, a target sample size of 367 patient participants was set. All clinicians who completed the survey in Stage 1 were invited to participate in the Stage 2 focus groups. Administrative staff who organised telehealth consults between March and December 2020 were recruited through convenience sampling to participate in semi-structured interviews. All participants provided informed consent prior to participation. A waiver from ethical review was provided by the institutional Human Research Ethics Committee (LNR/2020/QRBW/64414).

### Data collection

Stage 1 data was collected from September to October 2020. Patient survey questions included demographic and technology use information, as well as the Telehealth Usability Questionnaire (TUQ). The TUQ consists of 21 items covering six domains and is a reliable measure of users’ perceptions towards the usability of a telehealth system.^19^ A single open-ended question requested feedback on the challenges and/or enablers experienced when accessing care via telehealth. The clinician survey included demographic information and a series of closed- and open-ended questions regarding participants’ prior clinical exposure to telehealth, perceptions towards telehealth, as well as their preparation for and delivery of telehealth consults during the evaluation period. Items were drawn from the relevant published literature.^20,21^ Findings from survey responses informed the development of the semi-structured interview guides (clinician and AO staff) used in Stage 2 (December 2020–February 2021), which explored key factors influencing the implementation and sustainability of telehealth services. Interviews were undertaken by a member of the research team from an unrelated profession to the participants. All focus groups and 1:1 interviews were recorded (duration range: 9−53 min) and transcribed verbatim.

### Data analysis

Data collected from the patient and clinician surveys were collated and analysed separately. Quantitative data were analysed using descriptive statistics. For free-text responses, thematic analysis was employed as described by Braun and Clark.^22^ Themes were inductively derived from the data and then grouped as either challenges, enablers or strategies to the preparation and delivery of clinical care via telehealth. Qualitative data collected in Stage 2 was analysed separately using Template Analysis.^23^ A priori themes, based on the interview guides, were utilised as the initial coding template for each respective data set, and preliminary coding was undertaken using a thematic approach. Additional themes were generated for codes that did not readily adhere to a priori themes. To ensure the rigour of qualitative findings,^24^ a sample of transcripts were cross-checked for accuracy, while the consensus coding of each dataset was undertaken by a second investigator. Results from both stages are reported separately for each stakeholder group.

## Results

### Participant characteristics

Patients’ demographics and telehealth service information (n = 109, 30% response rate) are described in Table 1. Clinicians from six AHP departments participated in both survey and focus groups. Sixty-six clinicians completed the survey, identifying themselves as junior (n = 21), senior (n = 25) or advanced (n = 19) in their roles, with the majority being physiotherapists (n = 32, 48%). Most clinicians (n = 54, 84%) reported only delivering 1:1 telehealth consults, while 10 had conducted group sessions. Thirty-nine (59%) clinicians had provided care via telehealth prior to COVID-19. Twenty-four of the participating clinicians also attended the focus groups. Thirteen AO staff participated in interviews, including front-line staff (n = 11, two held telehealth specific roles) and team leaders (n = 2). All AO participants had experience supporting telehealth consults.

**Table 1.** Patient demographics and telehealth service information (n = 109).^a^

Parameters	n (%)
Gender	
Female	58 (53)
Male	51 (47)
Age (years)	
16–39 years	33 (30)
40–69 years	56 (52)
70 + years	19 (18)
Residential location	
Resides within local health service	64 (59)
Resides outside of local health service	45 (41)
Allied Health Service accessed via telehealth	
Physiotherapy	46 (42)
Nutrition & Dietetics	20 (18)
Occupational Therapy	17 (15)
Psychology & Neuropsychology	16 (15)
Speech Pathology	7 (7)
Social Work	3 (3)
Device used for telehealth appointments	
Laptop	35 (32)
Smartphone	29 (27)
Tablet	21 (20)
Desktop	23 (21)
Patient location for telehealth appointments	
Private residence	96 (88)
Work	6 (6)
Healthcare facility	6 (6)

^a^ Responses to survey items were optional.

### Patients’ experiences of AHP telehealth services

Patients rated their telehealth experience highly on the TUQ domains of usefulness, ease of use, effectiveness and satisfaction, with a lower rating for reliability (Table 2). Nearly all patients reported that the organisation of their telehealth consult/s was either ‘good’ or ‘very good’ (n = 105, 96%) and that they had access to an appropriate physical space (n = 106, 97%) and necessary equipment (n = 102, 94%). Most patients reported that they received adequate support to manage technical difficulties (78%) and would like to be offered telehealth for future AHP care (n = 85, 78%).

**Table 2.** Patient responses to the Telehealth Usability Questionnaire (TUQ) (n = 109).

Domain	Question	Median score^a^ (IQR) for question	Median score^a^ (IQR) for domain
Usefulness	Telehealth improves my access to healthcare services	6 (2)	6 (1)
	Telehealth saves me time travelling to a hospital or specialist clinic	7 (1)	
	Telehealth provides for my healthcare need	6 (2)	
Ease of use	It was simple to use the telehealth system	6 (1)	6 (1)
	It was easy to learn to use the telehealth system	6 (1)	
	I believe I could become productive quickly using this system	6 (2)	
	The way I interact with the telehealth system is pleasant	6 (1)	
	I like using the telehealth system	6 (2)	
Effectiveness	The telehealth system is simple and easy to understand	6 (1)	6 (1.5)
	This system is able to do everything I would want it to be able to do	6 (1)	
	I can easily talk to the clinician using the telehealth system	6 (1)	
	I can hear the clinician clearly using the telehealth system	6 (1)	
	I felt I was able to express myself effectively using the telehealth system	6 (1)	
	Using the telehealth system, I can see the clinician as well as if we met in person	6 (1.25)	
Reliability	I think the visits provided over the telehealth system are the same as in-person visits	5 (3)	4.7 (1.7)
	Whenever I made a mistake using the system, I could recover easily and quickly	6 (2)	
	The system gave error messages that clearly told me how to fix problems	4 (2)	
Satisfaction	I feel comfortable communicating with the clinician using the telehealth system	6 (1)	6 (1.5)
	Telehealth is an acceptable way to receive healthcare services	6 (2)	
	I would use telehealth services again	6 (2)	
	Overall, I am satisfied with this telehealth system	6 (1)	

IQR: interquartile range.

^a^ Responses provided on a 7-point Likert scale (1 = strongly disagree to 7 = strongly agree).

The analysis of respondents’ free-text responses (Table 3) identified four themes. Firstly, patients described their *experience of telehealth as largely positive*, as it provided them with a safe and convenient option to continue their care during COVID-19 restrictions. For many, it also highlighted the additional benefits of time and cost savings that telehealth affords. Secondly, many expressed that they felt telehealth was not suitable for all AHP consults, particularly for those that required a physical examination, and therefore would *prefer a blended model of telehealth and in-person consults*. With regard to *technology,* patients were overall comfortable in using the telehealth platform, although some reported that the *quality of the connection impacted their consult*. Finally, increased *support is required to engage with telehealth*, particularly for those patients who required assistance/support to participate fully in consults, while access to consistent technical support was required for both patients and clinicians.

**Table 3.** Patient perceptions of factors influencing telehealth implementation and sustainability (n = 74).

Themes	Sub-themes	Examples of participant quotes
Experience of telehealth was overall positive	Grateful for the opportunity to continue access to care during COVID-19 restrictions.	‘I really appreciated having the telehealth option because I was badly injured and needed to continue my physio during the COVID shut down and using telehealth meant I could do that’. [P36]
	Telehealth service provided convenience, reduced waiting and travel time and reduced cost	‘On top of the fact that I don't have to travel 250 km round trip and don't have to pay $80 in parking fees for a short appointment. Improved my mental health in many ways’. [P1]
	Avoiding hospital when immunocompromised reduced anxiety and supported feeling of safety during COVID restrictions.	‘Eliminated the need to go and visit the hospital – [I’m] a vulnerable patient with cancer history so have to be particularly careful with exposure’. [P18]
A blended model of telehealth and in-person consults is preferred	Preferred mode of delivery is dependent on appointment type or nature of tasks required for that appointment.	‘If I need to have an L-Dex reading or my measurements taken, a face-to-face appointment is needed. I would like to have some of my services via telehealth and some face to face when required’. [P3]
	Having the choice of a mix of in-person and telehealth appointments is preferred.	‘I would like to see it used more in follow up visits after initial face to face meetings’. [P48]
		‘Being pregnant it was a great way to communicate with my midwife or dietitian in the comfort of my own home but sometimes I did prefer a face-to-face appointment especially for check-ups in my second trimester’. [P80]
Technology	Telehealth platform was easy to use	‘Once the technical side was set up it is quite efficient’. [P19]
	Inconsistent telehealth connection impacted experience	‘Depending on internet at the time there could be a lag which would make it difficult. And sometimes the clinician was blurry’. [P81]
		‘Telehealth is difficult to pick up on body language and slight delay between message between patient and clinician. You can communicate but not as good as [in-person]’. [P34]
Support is required to engage in telehealth	More technology support is required	‘Needs better tech[nical] support on the hospital end’. [P23]
	Patient support is required to engage with telehealth	‘I am the carer for my husband who has dementia. There would be no way he could have joined telehealth sessions without assistance at home’. [P96]
	Patient support is required to prepare for and engage in telehealth consults	‘When I [practice] walking on my [prosthetic] leg, if I have the tablet just propped against something, then the clinician can't see me because I go out of camera view, and so it is a bit hard for the clinicians to be able to see me at all times’. [P42]

### Clinicians’ experiences of AHP telehealth services

The majority of clinicians agreed that there was value in using telehealth in their field of clinical care (92%), that telehealth was a legitimate part of their role description (89%) and that they would continue to support the ongoing use of telehealth (92%). Areas of greatest challenge included access to technical support, their lack of confidence in trouble-shooting technical difficulties, the suitability of the patient's physical environment when conducting telehealth consults and the patient's preparedness for the telehealth consult (Table 4).

**Table 4.** Clinician experiences providing care via telehealth (n = 66)^a^ using 5-point scale (Never to Always).

	Never/Rarely	Sometimes	Most times/Always
Question	n (%)	n (%)	n (%)
The physical infrastructure (e.g. physical space, telehealth equipment, etc.) within my department was readily available for when I had scheduled telehealth appointments	5 (8)	20 (31)	40 (61)
I had access to support when technical issues arose during a telehealth appointment	11 (17)	22 (35)	30 (48)
I felt confident trouble-shooting technical difficulties the patient was experiencing	15 (23)	34 (52)	16 (25)
The telehealth system was easy to use	3 (5)	15 (24)	46 (71)
I could develop an appropriate level of rapport with patients during the telehealth appointment	5 (8)	14 (22)	45 (70)
Patients were adequately prepared for their telehealth appointments (e.g. appropriate physical space, therapy equipment available, etc.)	11 (17)	26 (41)	27 (42)
The patient's physical environment prevented me from conducting an adequate consultation	21 (33)	29 (45)	14 (22)
I was able to complete my planned intervention within the allocated appointment time	8 (12)	8 (13)	47 (75)

^a^ Responses to survey items were optional.

Qualitative data highlighted seven themes that influenced the implementation and sustainability of telehealth (Table 5). Firstly, clinicians accepted *telehealth as a valid service delivery model*, and for many, it opened their eyes to its use for delivering a range of clinical services. However, telehealth was not considered suitable for every patient or clinical service, and therefore, the service mode (telehealth/in-person) needed to be tailored to the individual patient needs/condition. Secondly, access to *technological and physical infrastructure* influenced the ability to undertake some telehealth consults. While clinicians reported access to technological equipment had improved since the onset of COVID-19, equipment was still being shared between some staff, making it difficult for concurrent telehealth consults to occur. Clinical rooms also need to be ‘telehealth-ready’, as conducting clinical consults in a shared office space that lacked privacy was not appropriate. Thirdly, more *training in telehealth technology* was recommended. Although most clinicians acknowledged that telehealth platforms were easy to learn and operate, some had limited time for, or access to, training, which impacted confidence and the optimal use of the platform. Formal training for all staff in the telehealth platform was suggested, and ‘refresher’ sessions completed as needed. The *quality of the telehealth connection* was also viewed as an ongoing and variable issue. Clinicians described this as impacting the feasibility and effectiveness of the consult, and some clinicians felt ill-equipped to manage technical issues occurring at the patients’ end. Work shadowing/observation was considered a valuable activity for learning to navigate the telehealth platform and manage technical issues.

**Table 5.** Clinician perception of factors influencing telehealth implementation and sustainability (n = 24).

Theme	Sub-themes	Examples of participant quotes
Acceptance of telehealth as a valid service delivery model	In-person services converted to telehealth continue	‘We were doing 100% during COVID [mandated physical distancing restrictions] and it was successful. It was very successful as long as the connection was fine … I completed, you know whole therapy blocks that way and … we continue to do that’. [AHP66]
	Telehealth is not suitable for every patient	‘Because it [clinical caseload] varies so much, and our role is so hands on …[telehealth] does have a role and with COVID it's opened my eyes to using it more. But it couldn't take over’. [AHP44]
	Preference for initial assessments to completed in-person	‘So you might see them face-to-face … initially and then review them [via telehealth] … we’ve already done all those physical checks and I feel more confident to just do a progress check, so long as there are no new symptoms’. [AHP42]
Technology and physical infrastructure	Every space needs to be ‘telehealth-ready’	‘I like sitting in the [clinic] room because that equipment seems to stay there permanently’. [AHP64]
	Consults should be conducted in a private environment with minimal distractions	‘I get that we share space … but when you’re on a telehealth consult with [your] patient, and the person beside you is discussing care of a different patient’. [AHP22]
Training to use telehealth platform	Telehealth platform was easy to use	‘The program was easy to use and required very little training to be able to use independently’. [AHP12]
	Training provided was variable	‘Speed with which I had to learn the program - 5 min - prior to seeing a [patient] … with no chance to experiment and check that I knew how to use it’. [AHP16]
		‘She [telehealth champion] provided us with lots of PD during COVID so that helped a lot and … orientating to each telehealth system’. [AHP66]
	Training should be provided to all staff	‘When you start with the department or come back from [extended] leave … also if you’re changing workloads. If telehealth is a part of that, then part of that handover is orienting to that specific technology in that clinic’. [AHP50]
Quality of the connection and troubleshooting technical issues	Technical issues are ongoing	‘I think it's an ongoing challenge … but the quality of the connection was definitely poorer during COVID-19 [mandated physical distancing restrictions] than compared to when we continued to use telehealth’. [AHP66]
	Impacts the effectiveness of the consultation	‘We had a lot of technical difficulties which were a barrier to an effective clinical assessment’. [AHP42]
	Lack of confidence troubleshooting technical issues	‘So if I was doing a telehealth appointment on the day where [name of AO] wasn't there, I’d be really nervous’. [AHP62]
Ability to adapt clinical interventions	Adaptation is dependent on intervention/task with need for direct physical contact more challenging	‘Well, a lot of our work is education. So that was easy to transfer over to telehealth. But obviously, we can't do our [internal] pelvic floor examinations via telehealth at all’. [AHP43]
	Confidence in adaptation influences clinical judgement and perceived treatment effectiveness	‘Doing a neurological examination is an essential part of screening many of our patients. In most cases I felt unconfident in my diagnosis via telehealth because I was not able to perform a complete examination’. [AHP42]
	Evidence-based telehealth models supported adaptation	‘I think in clinical areas it works really well is like … a swallow therapy program or exercises working on their tongue or on their jaw opening, you can manage that really well by telehealth in terms of progressing the therapy.. or doing a swallow assessment…’. [AHP63]
	Support from telehealth mentor was beneficial	‘… I received a lot of training and mentorship… I wouldn't want a person to have any less. What I thought I received was good’. [AHP66]
	Practical training from clinical mentor is needed	‘A workshop where you can practice with a colleague as a mock patient’. [AHP25]
Patient selection and engagement	Willingness to engage with telehealth	‘On the ward and I’m often booking their telehealth for when they’re discharged, that's when they’re super keen. Because they’ve met us, I’ve already started [their therapy] … you have started a relationship’. [AHP62]
	Supporting patient specific needs	‘Our population group have difficulty communicating and there is a strong reliance on physical cues … this was difficult to do over telehealth, particularly if there was a poor internet connection’. [AHP08]
	Patients’ physical environment impacted consult	‘I had times when patient is at home, but because a lot of people were at home as well [during COVID-19 mandated physical distancing restrictions] … they might be doing the mowing next door or cutting some trees, and of course that affected our session’. [AHP61]
		‘…no matter how many times you tell someone, you must be somewhere private, most of them are okay but there are times where someone is driving or at the cafe’. [AHP59]
Administrative staff support	Staffing needs to support service demands	‘Having to do the bookings/re-bookings ourselves, organising patient login and sending the email etc. This ate up a lot of the clinical time’. [AHP24]
	Designated staff means that administrative tasks are more likely to be completed	‘I think what works really well … is having one key person, and that's their one role to do’. [AHP62]
	Adequate support directly influences an AHP’s decision to offer telehealth consults	‘I’m going to stop all of my telehealth programs … because I haven't got that support to help the patients connect and do that test connection, which is fundamentally necessary’. [AHP53]

The fifth theme is related to clinicians’ *ability to adapt clinical interventions for telehealth*. Clinicians described that while talk-based interventions were relatively easy to adapt, interventions that required physical contact were more challenging. For those tasks where adaptation had not been formally established, many clinicians lacked confidence in doing this and some questioned their clinical judgement and treatment effectiveness. Dedicated training was needed to develop clinical telehealth skills, and those working in departments with existing telehealth services had benefited from training/support from a colleague with telehealth expertise. Several *patient factors* were also viewed as key features influencing telehealth services. The consideration of functional status (e.g. level of physical, cognitive and communication functioning) and available supports was needed to ensure that patients were suitable to engage in telehealth consults, as was the appropriateness of the patient's physical location and surroundings to enable full participation in consult tasks. Patient willingness to engage in telehealth was also perceived as influencing consult success. Finally, *AO staff support* was critical in influencing telehealth services. While AO support had improved since the initial COVID-19 restrictions, many clinicians felt that even more was needed to match growing service demands. Having dedicated AO telehealth staff also meant that non-clinical tasks were more likely to be completed, and for some clinicians, this directly influenced their decision to offer telehealth to their patients.

### Administrative staff experiences of AHP telehealth services

Overall AO staff valued telehealth for the benefit it afforded patients to access care at a reduced cost and greater convenience while also reducing the health risk from COVID-19. The analysis of AO staff data identified four themes (Table 6) that influence telehealth services. Firstly, relating to the *AO workforce*, increased staffing was needed to meet growing telehealth service demands. While dedicated telehealth roles were required to support workflow, all AO staff should be able to organise telehealth bookings and provide basic technical assistance. Secondly, all work areas should be equipped with the necessary *technology and infrastructure* to support telehealth tasks, while IT systems need to be integrated to streamline patient information and appointment scheduling. Staff suggested that using a consistent telehealth platform would assist workflow. Thirdly, staff requested *training in telehealth*, suggesting that it should be part of mandatory training. There was a preference for written manuals and trouble-shooting guides coupled with practical training opportunities. Lastly, AOs described inefficiencies around the current *triaging and booking processes*, reporting that increased time was required when organising telehealth consults. Suggested improvements were consistent screening process, utilising existing IT systems to identify telehealth suitable patients and confirming which clinical services/consults could be offered via telehealth.

**Table 6.** Administration staff perception of factors influencing telehealth implementation and sustainability (n = 13).

Theme	Sub-themes	Examples of participant quotes
Administration officer workforce	More staffing required to meet service demands	‘Just making sure that if the telehealth is going to grow that the admin support grows with it’. [AO6]
	Takes longer to book telehealth appointment than in-person appointment	‘.. it takes maybe 15–20 min …to get a telehealth [consult] booked. It takes me about 17 s to book a face-to-face’. [AO6]
	Dedicated telehealth administration staff are needed to support workflow	‘I do think there should be specialized telehealth staff… around patient contact and around technology…to troubleshoot and be able to make sure the patient is set up properly’. [AO12]
	Need adequate telehealth trained staff to manage routine staff changes	‘When I have a day off here and there, there is no one that steps in … I’m sure they could fumble their way through… and I do have a procedure manual… so there's a loose succession plan’. [AO12]
	All administration staff should be capable of making telehealth bookings	‘… I feel like we should all be able to do it, if we’re trained in it. Like I feel it would be simple’. [AO7]
	All administration staff should be capable of providing basic IT troubleshooting	‘If it's something simple … we should be able to help the patient, or it would be good to know some basic strategies. And then if we can't do that then to escalate it up to the IT [department]’. [AO8]
Technology and infrastructure	All work areas need to be equipped with appropriate infrastructure	‘So we use the telehealth portal but we don't use virtual clinics because we don't have enough computer screen’. [AO2]
	Need consistent telehealth platforms across AHP	‘To simplify the process so that across all of Allied Health we’re all using a similar platform, I think that would be a great help’. [AO12]
	Integration of IT programs would improve workflow	‘It would be good to have a streamlined process across the whole of allied health …the platform being used, how patient data is being held’. [AO12]
	Notification within IT systems of patients suitable for telehealth	‘It would be good if we could have an indicator to see if they’re suitable for telehealth…so if there's a way we could have some sort of section in [online booking system] saying telehealth, that would be really good’. [AO11]
AO training	Telehealth training should be included in mandatory administration staff training	‘A uniform approach would be of benefit then everybody received training’. [AO12]
	Written manuals and telehealth troubleshooting guides	‘Quick reference guides would be good … because you forget the tricky little bits between consults’. [AO8]
	Practical telehealth training incorporating simulation	‘So a bit of one on one or a bit of simulated training…like to connect to a telehealth, do a test booking…’. [AO6]
	Refresher training should be available	‘… if they’re not in it everyday they get a refresher, once every six months or so’. [AO12]
Triaging and booking telehealth appointments	Time inefficiencies contacting clinicians to triage appointments	‘I would check with the clinician to see if it's appropriate and then I would change it … and then I would call the patient back and then you would have to pass the details onto the [telehealth AO] who books and sends the telehealth emails. It's a long process’. [AO4]
	Screening process required for telehealth appointments	‘It would make it a lot easier if there was some sort of indicator that telehealth was appropriate for that appointment’. [AO9]
	IT systems that support triaging	‘If there was a way we could visually see somewhere in the scheduling, if this patient is eligible for telehealth or not. And then that would save us double checking with the clinician’. [AO11]

## Discussion

The rapid adoption of telehealth to support the delivery of healthcare during COVID-19 has meant that usual service implementation processes could not be followed. Despite these challenges, the overall experiences of telehealth for the participants in this study were positive. Patients valued telehealth with almost 80% wanting to be offered future appointments, and going forward, both patients and clinicians prefer a blended model of telehealth and in-person care. Knowing this preference, the challenges identified during rapid implementation need to be addressed, so telehealth services can be embedded as standard care and be adapted to meet the changing needs of a post-pandemic world.

While existing AHP telehealth models and expertise were shared between departments to assist with rapid establishment, clinician and patient experiences highlighted that telehealth alone could not support all AHP clinical services or patient conditions. With the evidence base growing, the increasing awareness about validated telehealth models, along with guidance on how to effectively adapt these models, is needed. In the absence of published evidence, new AHP telehealth models should be established using patient-centred design principles.^25–27^ This will ensure that clinical tasks are translated safely and effectively into a remote environment supported by suitable technology and equipment. This process also requires the analysis of the patient’s functional status, support needs and physical environment. This is critical for home-based consults, where clinicians need to work with the patient in adapting their surrounding environment to maximise participation. Guided by these principles, encouraging service providers to be flexible and responsive in their telehealth service design will develop models that provide high-quality clinical care, meet patient needs, contribute to the evidence base and support service sustainability.

With the implementation of any service re-design, appropriate infrastructure and clear and streamlined workflow processes are needed to maximise service efficiency and overall user experience.^28^ Consistent with telehealth literature, relevant technology integrated into existing clinical spaces was viewed as fundamental to embedding telehealth as usual practice, while the interoperability of platforms with electronic medical records and scheduling systems was needed to improve workflow.^29^ Appointment of dedicated telehealth positions was suggested to facilitate service coordination and provide technical support to both staff and patients.^30^ Participants identified that telehealth consults were clinician-directed and required approval prior to scheduling. This necessitated multiple contacts between AO staff, patients and clinicians, decreasing the motivation to offer telehealth consults for some staff. Achieving consensus on which patient conditions/clinical services can be supported via telehealth will streamline workflow and promote the integration of telehealth models within AHP clinical care pathways.

While both patients and clinicians reported that the telehealth platforms were considered easy to navigate, technical issues were a key frustration. Technical disruptions are evitable in telehealth services, and for many of these issues, their resolution may be out of our control (i.e. poor bandwidth/connection). Telehealth participants need build tolerance to these disruptions and learn to adapt when it is safe and appropriate to do so, to optimise the clinical consult (e.g. the use of online store-and-forward of images in response to poor bandwidth connection). All participant groups lacked confidence in managing technical disruptions. Dedicated staff training and patient education are required to build skill in basic technical troubleshooting. Service providers also need to invest in responsive technical support, ideally from a nominated staff member, who is available to provide on-demand assistance to patients and clinicians.

While training in telehealth technology is essential to optimise its functionality,^29^ clinicians also need to learn to adapt their clinical and communication skills to deliver care and achieve therapeutic alliance in a remote environment.^13^ This study highlighted that for many physical tasks, clinicians become reliant on the patient to provide information that would normally be derived through touch, and/or non-verbal communication, which can be hindered if the patient is not within full view or the connection is poor.^31^ Structured, evidence-based theoretical and skill-based training is required for clinicians to achieve competency and confidence in delivering clinical care remotely and to learn how to adapt these skills within the changing virtual environment.^21,32,33^ Access to ongoing professional development and workplace mentoring is needed to maintain these skills.^33^ Digital health also should be embedded as core training within all pre-registration qualifications to ensure graduates are ‘telehealth-ready’ and positively influence the adoption of telehealth by the AHP workforce.^29,33^ Of equal importance is the need to provide patient education, not only in the use of technology but also in the preparation for their appointment, so they receive the maximum benefit from the telehealth consult.

A few limitations are acknowledged. While the survey response rate was lower than expected, all AHP departments were represented in patient and clinician responses. This study examines a large cohort of stakeholders associated with the AHP departments of a publicly funded quaternary facility. Hence, findings may not be generalisable to other facilities, services provided by other professions or privately funded models.

Telehealth enabled the continuity of care for AHP services in the face of a global pandemic. Its rapid implementation raised many challenges, and while study participants viewed telehealth positively, several key improvements were identified as being vital to service enhancement and sustainability. These were the development of new and adaptive AHP telehealth models, improved workflow processes, enhanced technical support and workforce and patient training. These findings are informing the next stage of this research, which aims to develop a strategic plan to enhance and sustain AHP telehealth services.
